# Challenges of the COVID-19 pandemic on women’s HIV harm reduction centers: a mixed-methods study

**DOI:** 10.1186/s12954-024-01060-4

**Published:** 2024-07-24

**Authors:** Azam Rahmani, Maryam Janatolmakan, Elham Rezaei, Leila Allahqoli, Arezoo Fallahi, Elham Ebrahimi, Mahnaz Motamedi, Fatemeh Yousefi, Serap Ejdar Apay

**Affiliations:** 1 Nursing and Midwifery Care Research Center, School of Nursing and Midwifery, Tehran University of Medical Sciences, Tehran, Iran; 2 Social Development and Health Promotion Research Center, Health Institute, Kermanshah University of Medical Sciences, Kermanshah, Iran; Nursing and Midwifery Care Research Center, Tehran University of Medical Sciences, Tehran, Iran; 3 Midwifery department, School of Nursing and Midwifery, Tabriz University of Medical Sciences, Tabriz, Iran; 4 Midwifery Department, Ministry of Health and Medical Education, Tehran, Iran; 5 Social Determinants of Health Research Center, Research Institute for Health Development, Kurdistan University of Medical Sciences, Sanandaj, Iran; 6 Department of Reproductive Health Midwifery, School of Nursing & Midwifery Nursing and Midwifery Care Research Center, Tehran, Iran; 7 HIV/STIs Department, Communicable Disease Control Center, Ministry of Health and Medical Education of Iran, Tehran, Iran; 8 Department of midwifery, reproductive health promotion research center, Ahvaz Jundishapur University of Medical Sciences, Ahvaz, Iran; 9 Faculty of Health Sciences, Atatürk University & Atateknokent Company, Erzurum, Turkey

**Keywords:** HIV, Harm reduction, COVID-19, Mixed method, Drop in center, High risk women

## Abstract

**Background:**

The COVID-19 pandemic has posed significant challenges to nations worldwide, affecting various sectors of society. Women’s HIV harm reduction centers, which provide critical services, have also been affected by these difficulties. This study aimed to examine the challenges of the COVID-19 pandemic on the services offered by women’s HIV harm reduction centers from the perspective of clients.

**Methods:**

A convergent mixed-method design was utilized to gain in-depth insights into the challenges of COVID-19 on the services provided by women’s HIV harm reduction centers, counseling centers, and night shelters that cater to women at risk, such as drug users, sex workers, and the homeless population, in three provinces (Tehran, Khuzestan, and Kermanshah) in Iran. The study was conducted from January to May 2023. The quantitative aspect of the study employed a cross-sectional method with a sample size of 430 individuals. A researcher-developed questionnaire was used to assess a range of services. The qualitative part of the study involved traditional content analysis and included 32 individual interviews. The integration of qualitative and quantitative results was performed during the interpretation phase to provide a comprehensive understanding of the challenges of COVID-19 on women’s HIV harm reduction centers.

**Results:**

In the quantitative phase, the mean age of women was 39.0 ± 10.2 years. 165 women reported a history of COVID-19, which accounts for 38.4% of the total. More than half of them (*n* = 102, 61.8%) recovering at home. The majority of women (*n* = 365, 84.9%) mentioned receiving the COVID-19 vaccine. COVID-19 diagnostic tests were conducted for 74.2% (*n* = 319) of women. Women expressed higher satisfaction with the services (HIV prevention services, and accommodation services) before the COVID-19 pandemic compared to the satisfaction during the pandemic. The qualitative analysis identified emerging challenges related to the COVID-19 pandemic in two categories: personal challenges and mismanagement of services, comprising nineteen subcategories.

**Conclusion:**

The findings of this study highlight the adverse impact of the COVID-19 pandemic on the services provided by women’s HIV harm reduction centers, in contrast to the pre-pandemic period. To mitigate these negative effects, it is crucial to implement preventive measures and practical solutions. This may involve addressing the personal and management challenges of the centers.

## Introduction

COVID-19 has emerged as a global health crisis, causing significant repercussions in various domains of countries, including healthcare, politics, society, education, and culture [1]. However, the impact of the pandemic has been diverse across these different levels [2, 3]. One area that has been profoundly affected is the implementation of HIV prevention and harm reduction programs worldwide. These programs aim to mitigate the risks associated with HIV at the community level, encompassing activities such as HIV testing, needle and syringe exchange programs, condom distribution initiatives, and HIV/STI counseling services [4, 5]. The beneficiaries of these programs often belong to vulnerable and marginalized sections of society, and their previous high-risk experiences make them particularly susceptible to even slight fluctuations in health and economic conditions [6, 7]. Global research has demonstrated the consequences of the COVID-19 pandemic on Syringe Service Programs (SSPs) [8, 9], condom distribution efforts [10, 11], and the availability of diagnostic tests for hepatitis viruses and HIV [4, 8]. For example, a study conducted in Ireland revealed the negative impact of the pandemic on women’s HIV harm reduction centers and the well-being of injecting drug users. These effects manifested as a decrease in the number of hepatitis and HIV tests conducted and restricted access to safe injection equipment [12]. Similarly, a study in Korea indicated a reduction in the proportion of individuals at risk of HIV infection and a significant decline in the utilization of hospitals and centers for HIV-related issues. Furthermore, a substantial majority of these individuals did not avail themselves of telehealth services during this period [13]. Another study conducted in Canada highlighted that approximately 48.6% of sex workers lacked access to government financial support throughout the pandemic [14]. In Iran, multiple studies have been conducted to assess the impact of the COVID-19 pandemic on harm reduction services. One study involved conducting individual interviews with a small number of drug users to gather insights into their experiences [15]. With a prevalence rate of 1.6–1.9% for HIV in Iran [16, 17], another study focused on examining the effects of the pandemic on the provision of HIV/AIDS-related services. This study sought the perspectives of policy makers, service providers, and researchers to gain a comprehensive understanding of the challenges faced [18]. Additionally, there was a study that specifically addressed the challenges encountered in providing COVID-19 prevention services to homeless drug users [19]. The disruptions in service delivery and limited access to essential resources have indeed heightened the vulnerabilities of marginalized populations in Iran. It is crucial for governments, healthcare providers, and organizations to address these challenges and ensure the continuity of services during and beyond the pandemic. While research in Iran has focused on homeless individuals and drug users, with qualitative approaches and presenting their viewpoints [15, 18, 19], there has been limited attention given to the impact of COVID-19 on the services provided by women’s HIV harm reduction centers, particularly when comparing the pre-pandemic period to the pandemic period. Therefore, this study was conducted to examine the specific effects of the COVID-19 pandemic on the services offered by women’s HIV harm reduction centers in Iran. In this study, a comprehensive analysis was conducted to explore the challenges posed by the COVID-19 pandemic on the service delivery process of women’s harm reduction centers from the perspective of clients. The study provided valuable insight into the strengths and weaknesses of these services and identified areas that require improved management and corrective actions to effectively address similar challenges in the future.

## Materials and methods

### Study design and settings

This study employed a convergent mixed design, which integrated quantitative and qualitative methodologies to comprehensively assess and validate the findings [20]. By combining both approaches, a comprehensive understanding of the challenges of the COVID-19 pandemic on the service processes of women’s HIV harm reduction centers from the perspective of clients was obtained. Both the quantitative and qualitative stages of the research were conducted separately. The study collected samples from four counseling centers affiliated with the Iranian Ministry of Health and six Harm Reduction Centers/night shelters affiliated with the Iranian Welfare Organization. These centers were located in Tehran, Khuzestan, and Kermanshah provinces. The selection of these three provinces was based diversity of provided services and socioeconomic status of the areas. These provinces were identified as among the most vulnerable places in Iran, characterized by higher levels of socioeconomic challenges and a greater demand for women’s HIV harm reduction services, as indicated by the higher number of referrals. By selecting centers from these provinces, the study aimed to capture the experiences and perspectives of individuals accessing women’s HIV harm reduction centers in areas with greater vulnerability and higher service utilization. This approach ensured that the findings would be representative of the most affected regions and provide valuable insights into the challenges of the pandemic on the services provided by women’s HIV harm reduction centers in these particular areas.

### Participants and data collection

This research was conducted between January and May 2023. The inclusion criteria for both qualitative and quantitative parts of the study were willingness to participate in the study, having a history of receiving services from the centers before and during the COVID-19 pandemic, the ability to communicate verbally, and awareness of time and place.

#### Quantitative part

In the quantitative section, participants who failed to complete at least 10% of the questionnaire questions were excluded from the study. We applied consecutive sampling for quantitative part and purposive sampling for qualitative part. To determine the sample size for the quantitative section, Cochran’s formula was used, resulting in a minimum required sample size of 340 participants. Considering potential attrition and incomplete questionnaires, the sample size was increased to 450 individuals. After removing the exclusions, 210 participants from Tehran, 110 from Khuzestan, and 110 from Kermanshah (430) participated in the quantitative section. Participants were asked to complete the questionnaire. To ensure consistency and minimize bias, the researcher took care to read the questions in the same manner for participants who were illiterate.

#### Qualitative part

In the qualitative section, participants were excluded if they were unable to recall certain services provided by the centers before and during the COVID-19 pandemic. We applied purposive sampling for qualitative part. For the qualitative part, a total of 32 women were included, with 12 participants from Tehran, 10 from Khuzestan, and 10 from Kermanshah. In the qualitative part of the study, data was collected through individual interviews. Focus group discussions were not feasible for this study due to several reasons. These reasons included limited active participation, as well as individual preferences for individual interviews; despite assurances of confidentiality, some women reported feeling uncomfortable speaking in a group setting.

### Study tools

#### Quantitative part

During the quantitative phase of the study, a questionnaire developed by the research team was administered to women attending HIV harm reduction centers. The primary objective of the questionnaire was to evaluate changes in service delivery before and during the COVID-19 pandemic. It was divided into three parts. The first part of the questionnaire aimed to gather personal information from the participants. This included details such as age, marital status, occupation, education, income level, duration of residence and contact with the centers, history of chronic diseases, previous COVID-19 infections, history of COVID-19 diagnostic tests, and information about COVID-19 treatment. **The second part** of the questionnaire consisted of 11 questions with yes or no options that assess the quantitative level of services provided before and during the COVID-19 pandemic. These questions addressed various aspects of service provision in harm reduction centers. The topics covered in this section included counseling services (such as psychology, midwifery, and general practitioner), HIV prevention education, HIV and hepatitis diagnostic testing, provision of condoms, syringes, and needle heads, quantity of accommodation-related services (such as sleep, food, and clothing), quantity of services related to COVID-19 prevention, and health services in the centers (including bathroom facilities and availability of detergents). These questions were administered both during the COVID-19 pandemic and before it, allowing for a comparison of service provision during the two periods. **The third section** of the questionnaire focused on assessing the quality of services provided in harm reduction centers. This part included inquiries about the quality of HIV prevention services, such as education, testing, and provision of condoms. It also addressed the quality of accommodation-related services, including sleeping arrangements, food, and clothing. Additionally, participants were asked to evaluate the quality of preventive measures taken during the pandemic. To rate these services, participants were provided with a four-point Likert scale, which encompassed the options of excellent, good, fair, or poor. To ensure the face validity of the researcher-developed questionnaire, a sample of 20 individuals was selected for testing. This process aimed to assess the questionnaire’s clarity and comprehensibility.

#### Qualitative part

In the qualitative part of the research, data was collected through semi-structured interviews. A total of 32 women agreed to participate in the interviews. The interviews focused on various key questions related to the impact of the COVID-19 pandemic on access to services and the participants’ quality of life. Some of the main topics covered during the interviews included changes in access to services, the effects of COVID-19 on quality of life (such as sleep, food, and overall health), strategies employed by participants to prevent COVID-19 infection and whether these strategies were followed, the impact of COVID-19 on access to food and other services (such as testing and screening), health-related risks experienced (both physical, psychological, and social) during the pandemic, changes in service delivery by center staff due to COVID-19, whether the centers have implemented COVID-19 management plans, unmet needs during the pandemic, and proposed changes to address the conditions arising from COVID-19. Additionally, exploratory questions were asked based on the participants’ responses to each main question, allowing for a deeper understanding of their experiences. Data collection continued until data saturation was reached. This means that interviews were conducted until no new information or insights could be gained from the participants.

### Data analysis

#### Quantitative part

Quantitative and qualitative data were collected simultaneously but analyzed separately in the study. For the quantitative analysis, the statistical software program IBM SPSS Statistics for Windows, version 19 (IBM Corp. 2012, Armonk, NY: IBM Corp) was utilized. Descriptive statistics such as mean (standard deviation) and frequency (percentage) were used to describe the quantitative variables.

#### Qualitative part

Regarding the qualitative data analysis, each individual interview was transcribed and analyzed in a step-by-step manner. Following the approach suggested by Granheim and Lundman [21], the entire interview or observation was considered as the most appropriate unit of analysis. Meaningful units, such as words, sentences, or paragraphs, were identified and categorized as semantic units based on their single meanings. Each meaning unit was then given a name corresponding to its explicit or implicit meaning. Categories were compared for differences and similarities, leading to the emergence of final themes. For the qualitative data analysis, the software Max-QDA version 20 was utilized.

#### Integration

The integration of the quantitative and qualitative findings took place during the interpretation stage of the study. This process involved examining consistent and contradictory data from both types of analysis. By considering the results of both quantitative and qualitative approaches, comparing the findings of the two sections with each other, and examining similarities and contradictions, a more comprehensive understanding of the research findings was obtained.

### Trustworthiness

Trustworthiness in the study was ensured through several measures. Firstly, the interview process was conducted by an expert with extensive experience in the field. This helped to maintain the quality and accuracy of the interviews. Secondly, five women who participated in the study were actively involved in the review process. They provided feedback on the codes extracted from their own interviews, which enhanced the credibility and trustworthiness of the findings. Furthermore, external researchers who were knowledgeable in qualitative content analysis independently examined and evaluated the codes and themes. This process of external validation added an additional layer of rigor and reliability to the study. To enhance the transferability of the findings, maximum variation sampling was employed. This sampling technique ensured the inclusion of women of different ages, locations within the city, and diverse socioeconomic statuses. It also encompassed various types of service recipients, such as sex workers, drug users, and injecting drug users. This approach allowed for a broader representation of the population and increased the generalizability of the findings to similar contexts.

### Ethical considerations

The research conducted in accordance with ethical guidelines and received approval from the ethics committee of Tehran University of Medical Sciences (IR.TUMS.FNM.REC.1401.175). All women who participated in the study were provided with a clear explanation of the study’s objectives. Informed consent was obtained from each individual, indicating that they understood the purpose of the research and voluntarily agreed to participate. To protect the participants’ privacy and confidentiality, they were assured that their personal information and discussions regarding the services provided by the centers would be kept confidential. Any identifying information was handled with strict confidentiality and was not disclosed in the study’s reporting or publication. Additionally, the women were informed of their right to withdraw from the study at any time if they chose to do so. They were assured that their decision to withdraw would not have any negative consequences, and their participation in the study was entirely voluntary.

## Results

The results of this study are presented separately in two parts: quantitative and qualitative.

### Quantitative results

The mean age of women who required services at the harm reduction centers was 39.0 ± 10.2 years. A total of 430 eligible women were assessed for inclusion in the study. Among them, 111 individuals (25.8%) had a diploma, and 40.5% (*n* = 174) were divorced. More than half of the women had children (*n* = 273, 63.5%). On average, the women had been in contact with women’s HIV harm reduction centers for 5.0 ± 3.0 years. Out of the total, 124 women (28.8%) were unemployed, and 31.6% (*n* = 136) had a history of underlying diseases (Table 1).

**Table 1:** Sociodemographic characteristics of the women (*n* = 430)

Variables			*N* (%)^a^
Age (Mean ± SD) ^b^			39.0 ± 10.2
Duration of contact with women’s HIV harm reduction centers (Year) (Mean ± SD)^b^			5.0 ± 3.0
Education	Illiterate		49(11.4)
	Elementary		98(22.8)
	Guidance		103(24.0)
	High school		33(7.7)
	Diploma		111(25.8)
	Associate degree		12(2.8)
	Bachelor’s degree		23(5.3)
	Master’s degree		1(0.2)
Marital status	Single		87(20.2)
	Married		96(22.3)
	Divorced		174(40.5)
	Widow		68(15.8)
	Temporary marriage^c^		5(1.2)
Have Children	Yes		273(63.5)
	No		157(36.5)
Job	Unemployed		124(28.8)
	Sex Worker		118(27.4)
	Cleaner		84(19.5)
	Housewife		68(15.8)
	Babysitter or elderly nursing		18(4.2)
	Street peddler		11(2.6)
	Waste collector		6(1.4)
	Beggary		1(0.2)
Underlying disease	Yes	Glands	54(12.6)
		Cardiovascular	10(2.3)
		Neurological	11(2.5)
		Skeletal and muscular	9(2.1)
		dermal	3(0.6)
		Psychological	25(5.8)
		hematologic	11(2.5)
		Respiratory	9(2.1)
		Internal	3(0.6)
		Immune system	2(0.5)
	No		294(68.4)

^a^ Number (percentage), ^b^ mean ± standard deviation, ^c^ It is a type of marriage in which the duration and amount of the woman’s dowry are determined in advance. In this marriage, a temporary agreement is not given to the woman for alimony, and the man and the woman do not inherit from each other,

Among the participants, 165 women (38.4%) reported a history of COVID-19 infection, with the majority experiencing it once (*n* = 129, 78.2%). The average duration of COVID-19 treatment was 8.1 ± 5.7 days. More than half of the women spent their recovery period at home or at a relative’s house (*n* = 61.8, 102%). The majority of women mentioned receiving the COVID-19 vaccine (*n* = 365, 84.9%), while some declined due to fear and rumors about vaccine side effects. Fifty-five point 1% of women (*n* = 201) had received both doses of the COVID-19 vaccine. Diagnostic tests for COVID-19 were conducted for 74.2% of women (*n* = 319), with 76.8% (*n* = 245) utilizing nasal swabs for the tests (Table 2).

**Table 2:** Distribution of the COVID-19 in women’s HIV harm reduction centers

Variables		*N*(%)^a^
Duration of treatment) Days) (Mean ± SD) ^b^		8.1 ± 5.7
Infected with COVID-19	Yes	165(38.4)
	No	265(61.6)
The number of times of infecting with COVID-19	Once	129(78.2)
	Twice	27(16.4)
	Three times	9(5.4)
Quarantine place	Home	102(61.8)
	Night shelter	32(19.4)
	Hospital	31(18.8)
History of receiving the COVID-19 vaccine	Yes	365(84.9)
	No	65(15.1)
The number of times of vaccination	Once	53(14.5)
	Twice	201(55.1)
	Three times	100(27.4)
	Four times	11(3.0)
The place of vaccination	City	244(66.8)
	Women’s HIV harm reduction centers	108(29.6)
	Hospital	5(1.4)
	Clinic	6(1.7)
	Prison	2(0.5)
Conducting a diagnostic test for COVID-19	Yes	319(74.2)
	No	111(25.8)
Type of diagnostic test for COVID-19	Nasal swab	245(76.8)
	Blood test	17(5.3)
	Lung X-ray	13(4.1)
	Nasal swab and lung x-ray	12(3.8)
	Nasal swab and Blood test	32(10.0)

^a^ Number (percentage), ^b^ mean ± standard deviation

The level of satisfaction among women with the service delivery of the centers was more favorable before the COVID-19 pandemic compared to during the pandemic (Table 3).

**Table 3:** The level of satisfaction of women with the way of providing service before and during the COVID-19 pandemic (*n* = 430)

Variables		Satisfaction level N(%)^a^			
		Excellent	Good	Average	Weak
The quality of HIV prevention services (education - HIV testing - providing condoms)	Before COVID-19^b^	241(56.1)	141(32.8)	35(8.1)	13(3.0)
	During COVID-19^b^	231(53.7)	140(32.6)	36(8.4)	23(5.3)
Quality of accommodation services (sleeping, food, clothing, etc.)	Before COVID-19	205(47.7)	175(40.7)	35(8.1)	15(3.5)
	During COVID-19	184(42.7)	174(40.5)	48(11.2)	24(5.6)

^a^ Number (percentage), ^b^ in the survey, we considered the COVID-19 pandemic period in Iran (2020 February − 2023 July), and the non-pandemic period (before December 2019, and from August 2023 onwards)

### Qualitative results

The qualitative part of this research involved the analysis of the interviews, which resulted in the identification of 818 codes. These codes represent emerging challenges related to the COVID-19 pandemic (Table 4).

**Table 4:** Themes, categories and subcategories of women’ views on the effects of COVID-19 on the services provided by women’s HIV harm reduction centers

Theme	Categories	Subcategories
Emerging challenges	Personal Challenges	Mental restlessness
		Improper Sexual Contact
		Problem of underlying disease
		Job loss
		Financial instability
		Substance Abuse
		Social exclusion
	Mismanagement of services	Non-free healthcare services
		Unfavorable accommodation
		Undesirable nutrition
		Low quality of education
		Unacceptable STDs* service
		Lack of financial support
		Lack of self-reliance program
		limited admission
		Inflexible rules
		HRC* deficiency
		Unrestricted for high-risk groups
		Lack of monitoring and inspection

*Sexually transmitted diseases, ** Harm reduction centers

### Emerging challenges

Two categories were identified under this theme: personal challenges and mismanagement of services.

#### Personal challenges

The overall health of a person is achieved when all of their physical, mental, psychological, financial, economic, work, sexual, social, and cultural needs are met. Any disruption in these areas can impact a person’s health. This was especially crucial during the COVID-19 pandemic, as elements such as mental restlessness, improper sexual contact, problem of underlying disease, job loss, financial instability, substance abuse, and social exclusion were highlighted in the participants’ statements.

#### Mental restlessness

Participants have expressed that feeling insecure and unnoticed disrupted their peace of mind, disappointment, and this has been particularly evident during the COVID-19 pandemic.

“*During the Corona*,* I rarely went out of the shelter due to the fear of contracting the virus. Inside the shelter*,* I did not feel relaxed because of washing the surfaces*,* inspection*,* and the noise of the others*.“P3.

“*I was a drug user and divorced a few years ago. I think loneliness and stress are bad for me! Because it unconsciously leads me to use drugs! During the time of Corona*,* I felt death closely because every day I heard the statistics of deaths and patients*,* my situation was awkward*,* and I was really under pressure. I wanted to talk to someone 24 hours a day*,* but everyone was affected by this virus*,* and no one paid attention to me*.“P9.

*“I committed suicide once in the past. During the Corona*,* I felt so bad that I wanted to kill myself again. Some of the people I loved died because of Corona. On the other hand*,* I had no job and no future! Since the morning when we were imprisoned here*,* neither fun nor variety! Damn Corona*,* go away and never happen again.” P27*.

#### Improper sexual contact

One of the target groups of women’s HIV harm reduction centers was sex workers who sometimes did not follow health recommendations. During the COVID-19 pandemic, the existence of risks due to not observing social distance with carriers or infected people made the behavior of this group more challenging. Many of their sexual partners did not agree to use protective measures such as the use of condoms and masks, therefore, this situation made them very worried.

*“They told us to wear masks and use condoms. Don’t have too much sex. Well… but where do we get our money from? Our customers do not accept such conditions and say that we are not satisfied like this*,* so what should we do?” P17*.

#### Problem of underlying disease

One of the concerns during the COVID-19 pandemic was the potential for an increased number of women with chronic conditions contracting the virus. This issue was identified as a challenge in the women’ statements.

“*I have knee arthritis. During the Corona era*,* I had to move a lot to maintain hygiene and wash my hands and face*,* and this was a challenge for me.” P13*.

“*I have diabetes. In Corona*,* I heard that diabetic patients should be more careful and comply more. It was really painful because I couldn’t do anything. I didn’t eat according to my illness*,* so I didn’t take care of myself like I should have. That time passed bitterly for me.” P26*.

#### Job loss

The majority of women in the study faced the challenge of unemployment, which was exacerbated by the COVID-19 pandemic. The implementation of mandatory quarantine and social distancing measures led to the closure of many women’s part-time jobs, creating a dual challenge for them.

*“I was a peddler in the subway*,* but I lost that job during Corona*,* and it was a big problem for me because I lost my income.” P16*.

*“I am a sex worker and earn my income from this job*,* but in Corona due to the fear of contracting the virus*,* I had quarantined myself in the night shelter*,* which was a big shock for me.” P22*.

#### Financial instability

During the COVID-19 pandemic, they indicated that their financial instability was a significant challenge.

“*During the Corona*,* my financial situation was not good at all. My income was from the salary from cleaning houses*,* which I lost it*,* due to the fear of the customers getting infected with Corona.” P18*.

*“ I am a poor person*,* but during the corona*,* due to the increase in the cost of food*,* clothing*,* and hygiene items*,* I felt poverty more.” P25*.

#### Substance abuse

One of the challenges faced by the women in this study was their addiction to drugs and alcohol, which was further exacerbated during the COVID-19 pandemic. Interestingly, there was an increase in their substance usage during this time, partly influenced by rumors circulating about the potential preventive effects of these substances in controlling the virus.

*“I personally struggled with alcohol addiction during the COVID-19 period. The advertisements promoting the benefits of alcohol disinfectants created a false sense of permission for me to consume more*,* leading to a significant increase in my alcohol consumption.” P4*.

#### Social exclusion

Women highlighted their lack of acceptance by society, which became more prominent during the COVID-19 pandemic, as many individuals viewed them as carriers of the virus.

“*Many of my old friends in Corona cut off their contact with me because they knew about my job as a sex worker and were afraid that they would get Corona from me. Being ignored by them was a big challenge for me.” P5*.

Numerous personal challenges suggest that crises like the COVID-19 pandemic can overshadow people’s resilience and patience when dealing with these issues, reducing their ability to cope. As a result, the lack of management can transform these challenges into daily concerns for individuals.

### Mismanagement of services

The lack of proper management of services in women’s harm reduction centers was the second category of challenges felt during the COVID-19 pandemic and was extracted from the statements of the participants. Subcategories included non-free healthcare services, unfavorable accommodation, undesirable nutrition, low quality of education, unacceptable STDs service, Lack of financial support, lack of self-reliance program, limited admission, inflexible rules, HRC deficiency, unrestricted for high-risk groups, and lack of monitoring and inspection.

#### Non-free healthcare services

One of the essential services provided to women in women’s HIV harm reduction centers is access to health and treatment services, which includes doctor’s visits and medication prescriptions. However, the fact that certain additional services are not offered free of charge can pose a problem for these women, considering their poor economic circumstances. This issue became even more significant during the COVID-19 pandemic when women who engaged in part-time or informal employment had to stop working, resulting in a complete loss of their already limited income.

“*The pharmaceutical and medical situation here did not change before and during Corona. The doctor prescribes for us*,* and we have to*.

*prepare it ourselves! It would be good if the medicine was provided by the center. Because at the time of Corona*,* there was not enough medicine*,* and it was difficult to access it*.“P30.

#### Unfavorable accommodation

Having suitable living conditions is a persistent concern for women in HIV harm reduction centers, and this concern was exacerbated during the COVID-19 pandemic due to quarantine conditions and health restrictions.

“*This center was much better in its normal condition*,* but during the Corona*,* the condition of the toilets and bathrooms became awful because everyone was afraid of catching the Corona and therefore*,* every one used the toilets and bathrooms too much. With this limited number of bathrooms and toilets*,* we had to stand in line for half an hour.”* P21.

#### Undesirable nutrition

One of the services offered by women’s HIV harm reduction centers is providing meals to the women. However, some participants mentioned that the quantity and quality of the meals sometimes did not meet optimal conditions. During the COVID-19 pandemic, this service experienced challenges in terms of quality and variety.

*“Before the Corona*,* the food was much better*,* but during Corona*,* the food quality was not good*,* or there was no variety.”* P15.

“*I always heard on TV that good nutrition is effective in preventing the Corona virus*,* but who listens? They gave us the same food with the same variety and bad quality.“*P22.

#### Low quality of education

One of the needs expressed by women in HIV harm reduction centers was their desire for education and training. Participants highlighted the importance of providing comprehensive training, particularly during the COVID-19 pandemic.

“*Unfortunately*,* during the time of Corona*,* our center taught us less*,* and we ourselves learned what to do from TV*,* the street*,* and the bazaar.”* P15.

#### Unacceptable STDs service

One of the key responsibilities of women’s HIV harm reduction centers is to provide tools for preventing high-risk behaviors, such as the delivery of needles, syringes, and condoms. However, according to the findings of this study, women reported a decrease in the availability and quality of condoms during the COVID-19 pandemic. Participants expressed that prior to the pandemic, the services provided by the centers were better, with more equipment and facilities available. However, during the pandemic and continuing to the present day, the number of condom packages received by the women has significantly decreased. Furthermore, they noted that the quality of the condoms provided has deteriorated, with reports of easily torn condoms.

“*Before Corona*,* the service was better because they had more equipment and facilities but in Corona and even now*,* the number of condom packages I get is very few and it doesn’t have good quality at all. Now they give one or two packs every few months. It wasn’t like that in the past*,* we used to come month after month and get a good number of condoms*.” P21.

#### Lack of financial support

Women’s HIV harm reduction centers often provide livelihood packages, including essential food items such as rice and meat. However, according to the statements of the women in this study, these supports have sometimes been reduced. Additionally, some women expressed the belief that they should receive a fixed monthly salary during the COVID-19 pandemic.

*“Before Corona*,* they gave us a livelihood package*,* especially to those who had children. In Corona*,* they didn’t give us a livelihood package anymore*,* I hope this will continue again and they will help us like this at least a few times a year.”* P12.

“*We are not in a good economic situation. We also lost our part-time job due to Corona. We really needed a source of support. It would be great if the government considered a fixed and partial source of income for us*.” P19.

#### Lack of self-reliance program

Although women’s HIV harm reduction centers do not primarily focus on empowering participants, the neglect of this issue emerged as a challenge during the COVID-19 pandemic in conversations among women in the study.

“*Before Corona*,* we worked in a sewing workshop inside the center. It didn’t pay us much*,* but it was good. I don’t know what happened in Corona*,* they changed the manager and he closed the workshop and we were unemployed. So*,* shouldn’t the organization have replaced him?” P14*.

“ *… I wish professional management such as hairdressing*,* nail art*,* etc. would provide us with the possibility to become competent in that profession after a while.” P23*.

#### Limited admission

During the COVID-19 pandemic, some women reported that certain centers were temporarily closed or had restrictions on accepting women, which created difficulties for them.

“*I changed two centers during Corona*,* one of them was closed*,* and the other one was limited due to the fear of Corona*,* and I had to come here.” P24*.

“*During the time of Corona*,* they checked us every day to make sure we were not infected*,* and they did not allow some women to enter*,* saying that you should go and get a health certificate.” P28*.

#### Inflexible rules

Women expressed the need for flexible rules to be implemented in their centers during the COVID-19 pandemic. One participant mentioned that the strict rules and restrictions made it difficult for them to freely engage in activities and even go outside. They expressed a desire for less strictness.

*“The biggest problem we had during Corona was that they didn’t let us do whatever we wanted to do in the center. Even if you just went out*,* you had to answer hundreds of people when you came back and get checked to make sure you didn’t catch the corona virus. I wish they weren’t so strict.” P19*.

#### HRC deficiency

Women noted that the number of women’s HIV harm reduction centers is very limited, and this problem was more critical during the COVID-19 pandemic than before because some centers were either closed due to health guidelines or accepted a limited number of participants.

*“During the time of Corona*,* a large number of womens lived in a limited place without facilities*,* with minimal social distance. How many of these centers are there in Tehran? Well*,* if the number increases*,* access to these centers will be better*,* and not too many people will enter one center and will be divided among the centers.” p21*.

#### Unrestricted for high-risk groups

One of the problems of women’s HIV harm reduction centers was the presence of a large number of individuals with a history of risky behaviors, which sometimes seem difficult to manage. Women reported that since during the COVID-19 pandemic the law of quarantine was observed, they were constantly present in the centers and sometimes women with risky behaviors caused problems for the staff and others.

“*During Corona*,* I didn’t go out and I was always in the night shelter. There were several women in our room who worked as sex workers. They went out every day and when they came back*,* it was clear how many people they had sex with! So why should I share a room with these women?” P10*.

#### Lack of monitoring and inspection

Monitoring the performance of employees at women’s HIV harm reduction centers is a responsibility of the upper-level managers, as highlighted by the women. However, they mentioned that this monitoring process was not always conducted adequately, particularly during the COVID-19 pandemic.

*“During the period of the COVID-19 pandemic*,* when officials visited the centers*,* they would distribute masks*,* and when they wanted to report on the services provided*,* they would take pictures of us. In my opinion*,* monitoring should focus on actually observing and evaluating the services being provided.” P29*.

During the COVID-19 pandemic, women highlighted several needs such as unlimited admission to women’s HIV harm reduction centers, as some centers were closed or had restrictions on accepting women. They also expressed the need for flexible rules within the centers to allow more freedom and less strictness. Furthermore, the women reported difficulties in managing high-risk groups within the centers during the pandemic, and they called for improved monitoring and inspection processes to ensure effective performance evaluation.

The challenges related to managing centers highlight the importance of having proper structures and rules in place to ensure their survival. These systems can face difficulties during crises such as COVID-19 when their usual processes are not executed properly. This is a stressful situation for the recipients of services from these centers.

## Discussion

This research investigated the challenges of the COVID-19 pandemic on the provision of services at women’s HIV harm reduction centers in Tehran, Khuzestan, and Kermanshah provinces from the perspective of the clients of these centers. In the qualitative phase of the study, the first category of emerging challenges extracted from the participants’ conversations was “personal challenges.” Subcategories of this class included mental restlessness, inappropriate sexual contact, underlying disease, job loss, financial instability, substance abuse, and social exclusion.

Certain women, particularly those engaged in sex work, expressed a preference for seeking accommodation at the homes or residences of friends instead of utilizing night shelters. Their decision was influenced by concerns related to their physical safety, given the nature of their work involving multiple sexual interactions. Additionally, these women mentioned a decrease in the frequency of leaving their homes and visiting women’s HIV harm reduction centers. The findings of a multinational scoping review study, which examined 63 articles, revealed diverse outcomes. The study’s investigations indicated that despite the challenges posed by the COVID-19 crisis, these women acknowledged continuing their activities [22]. The results of the current study reveal that several factors have contributed to women engaging in less risky behavior and visiting the centers less frequently. These factors include the women’s fear of contracting COVID-19, the closure of certain centers, extensive quarantine measures, mandatory social distancing, and extensive media coverage in Iran regarding the death and infection rates of COVID-19. A scoping review stud highlighted the significant effects of COVID-19 physical distancing measures on vulnerable population groups in society. These effects include long-term loneliness, psychological distress, unemployment, loss of income, food insecurity, widespread inequality, and disruption of access to social support and health services. The study emphasized that these unintended consequences of physical distancing have further exacerbated the vulnerabilities of these groups, underscoring the negative impact of such measures [23]. The qualitative findings indicated an increase in drug and alcohol use during the COVID-19 pandemic compared to before. This rise was attributed to people’s misconception that these substances could prevent the coronavirus, as well as the impact of quarantine measures leading individuals to seek solace in these substances due to compulsion and social isolation. The finding of a review study encompassing 27 studies conducted worldwide revealed a varied trend (increase, decrease, or no change) in drug use among individuals during the COVID-19 pandemic compared to before [24]. Also, Piccio et al. (2020) conducted a study in Spain and found that the incidence of substance abuse among their clients increased during the COVID-19 pandemic [4]. In our study, we found that participants faced financial problems during the COVID-19 pandemic. Another study in Canada highlighted how the government’s financial barriers reduced support for sex workers during this time. Consequently, the financial assistance for these individuals decreased during the pandemic [14]. Amid the issues faced by the participants in the current study due to the COVID-19 pandemic, a separate study in Iran found that policymakers, service providers, and researchers acknowledged the pandemic’s diverse impact on their lives. They noted facing mental health challenges, financial difficulties, changes in care plans, and alterations in the risk behaviors of their clients [18]. In the context of the personal challenges faced by the participants in the current study, Deilamizade, emphasized challenges including growing social isolation, inadequate shelter capacity, increasing social stigma, and marginalization of clients in harm reduction centers. These issues have created obstacles to delivering COVID-19 prevention services to homeless individuals with substance abuse issues [19]. It seems that clients at harm reduction centers in various communities have encountered diverse challenges due to the COVID-19 pandemic. These challenges are not specific to any particular country, and everyone has been affected in some way.

The second category of emerging challenges extracted from participants’ conversations was service mismanagement. Subcategories of this class include non-free health services, poor housing, poor nutrition, poor quality of education, unacceptable sexually transmitted disease services, lack of financial support, lack of self-reliance programs, limited admissions, inflexible rules, lack of human rights compliance, inadequate support for high-risk groups, lack of supervision and inspection.

The study’s findings indicated that women were more satisfied with the services provided by the centers before the COVID-19 pandemic compared to the period during the pandemic. These results align with previous studies that have also highlighted the adverse effects of the COVID-19 crisis on the quantity and quality of harm reduction services [4, 25, 26]. It appears that the country’s overall policies and organizational plans have been primarily focused on the COVID crisis management in response to the COVID-19 situation [27, 28]. Consequently, considerable resources and efforts have been diverted to the healthcare system, which has ultimately impacted the quality of services provided by women’s HIV harm reduction centers [5]. According to the perspectives of women in this research, women’s HIV harm reduction centers during the COVID-19 pandemic primarily prioritized implementing health protocols to prevent COVID-19 transmission, which resulted in reduced focus on harm reduction services compared to before the pandemic. A survey conducted in China during the pandemic revealed that a significant percentage of individuals living with HIV were at risk of discontinuing their antiretroviral therapy, and many women expressed uncertainty about accessing antiviral medications [29]. Multiple studies have also shown a decline in the quantity and quality of HIV-related services during the COVID-19 pandemic [4, 5]. During the COVID-19 pandemic, women have reported a decrease in the quantity and quality of tools, such as condoms, provided by women’s HIV harm reduction centers to prevent risky behaviors. A study conducted in South Africa revealed that around 25% of individuals encountered difficulties in accessing condoms [30]. This finding highlights the significant impact of COVID-19 on existing health interventions within society.

The findings of the current research, which comprised both quantitative and qualitative components, revealed that women expressed a demand for psychological counseling services from harm reduction centers during the Covid-19 outbreak, and these services were indeed offered. A qualitative study highlighted that sex workers experienced multiple stressors during the COVID-19 epidemic, and seeking support from social networks emerged as one of their coping strategies to navigate these challenges [31].

The findings of the current research, both quantitative and qualitative aspects, indicate that COVID-19 screening was conducted once or twice in all three provinces. A global survey conducted in 2021 across 77 countries, focusing on substance disorder treatment and harm reduction services, revealed that only 44.3% of respondents from high-income countries (*N* = 95), 32.2% from middle-income countries (*N* = 34), and 40.1% from low-income countries (*N* = 48) reported being screened or tested for COVID-19 and receiving a diagnosis [32]. Despite challenges such as limited diagnostic kits for COVID-19, resource and manpower shortages, high costs, economic sanctions, and a small number of women’s HIV harm reduction centers [33], Iran seems to have been able to provide satisfactory services in this field.

According to the results of our study, another challenge faced by the women was the changes implemented in the structural and supervisory aspects of the centers. However, other studies have shown that implementing changes in service delivery, such as virtualization of services and increased flexibility in opioid substitution treatment guidelines for vulnerable groups, have presented an opportunity to enhance service design and better address the diverse needs of vulnerable individuals [34, 35]. It seems that the clients of the harm reduction centers were not satisfied with the management and structural changes implemented during the COVID-19 pandemic. They perceive these changes as a challenge related to the mismanagement of the centers.

This research possessed two notable strengths. Firstly, it encompassed multiple centers across the country, providing a more comprehensive perspective. Secondly, it delved into specific sensitive issues within Iranian society. However, despite these strengths, the study also encountered certain limitations. One limitation was associated with the participants’ mental state during questionnaire completion. Although efforts were made to engage women at appropriate times, complete control over this factor was beyond the researcher’s control. Another constraint involved the challenge of accurately recalling service provision both before and during the COVID-19 pandemic. To address this issue, a specific time frame focusing on the spring and summer periods was implemented to enhance the accuracy of data collection. The study encountered a limitation in conducting analytical statistical tests across the included centers. This limitation arose from the diversity in service provision among different centers, including new establishments and changes in management. The centers were scattered in terms of the services they provided because the sampling was done from three types of centers: women’s centers affiliated with the Ministry of Health, harm reduction centers affiliated with Welfare Organization, and night shelters. These centers catered to different client populations. For example, some women’s centers affiliated with the Ministry of Health primarily served sex worker clients, while harm reduction centers covered a mixture of homeless women and substance abusers. Some of the centers were newly established or experienced changes in management, leading to a lack of written information on the access to services for the individuals served, hindering the possibility of comparing the two study periods. Due to these reasons, the information from all the centers was merged to provide an overview. Lastly, given that the descriptive results held higher statistical power than the analytical results in this study, it was preferred to report the findings descriptively.

## Conclusion

The COVID-19 pandemic has had detrimental effects on both the quantity and quality of services offered by women’s HIV harm reduction centers. The qualitative part of the research revealed individual and managerial challenges, highlighting the increased needs and difficulties faced by the women during the pandemic. It is recommended to develop strategies that address the negative impacts of the pandemic on service provision. One potential approach could involve prioritizing initiatives that provide women with stable employment opportunities, permanent housing options, and expanding the availability of mobile services. Future studies could evaluate the effectiveness and consequences of implementing these strategies to improve the service delivery process of women’s HIV harm reduction centers, particularly during times of crisis.

## Data Availability

No datasets were generated or analysed during the current study.

## Abbreviations

HIV: Human immunodeficiency virus
STDs: Sexually transmitted diseases
HRC: Harm reduction center
